# Fine-Mapping and Functional Analyses of a Candidate Gene Controlling Isoflavone Content in Soybeans Seed

**DOI:** 10.3389/fpls.2022.865584

**Published:** 2022-04-25

**Authors:** Ruiqiong Li, Jianan Zou, Dongming Sun, Yan Jing, Depeng Wu, Ming Lian, Weili Teng, Yuhang Zhan, Wenbin Li, Xue Zhao, Yingpeng Han

**Affiliations:** ^1^Key Laboratory of Soybean Biology in Chinese Ministry of Education (Key Laboratory of Soybean Biology and Breeding/Genetics of Chinese Agriculture Ministry), Northeast Agricultural University, Harbin, China; ^2^College of Tropical Crops, Hainan University, Haikou, China; ^3^College of Life Science, Huaiyin Normal University, Huaiyin, China

**Keywords:** soybean, isoflavone content, quantitative trait loci, *GmMT1*, disease resistance, stress

## Abstract

Isoflavones, one of the most important secondary metabolites produced by soybeans (*Glycine max* (L.) Merr.), are important for a variety of biological processes, and are beneficial for human health. To identify genetic loci underlying soybean isoflavone content, a mapping population containing 119 F_5:18_ recombinant inbred lines, derived by crossing soybean cultivar “Zhongdou27” with “Dongong8004,” was used. We identified 15 QTLs associated with isoflavone contents. A novel loci, qISO19-1, was mapped onto soybean chromosome 19 and was fine-mapped to a 62.8 kb region using a BC_2_F_2_ population. We considered *GmMT1* as a candidate gene for the qISO19-1 locus due to the significant positive correlation recovered between its expression level and isoflavone content in the seeds of 43 soybean germplasms. Overexpression of *GmMT1* in *Arabidopsis* and soybean cultivars increased isoflavone contents. Transgenic soybeans overexpressing *GmMT1* also exhibited improved resistance to pathogenic infection, while transgenic *Arabidopsis* resisted salt and drought stress.

## Introduction

Isoflavones, important secondary metabolites commonly known as phytoestrogens, are one of the most highly bioactive flavonoid classes (Wahajuddin et al., 2013). Isoflavones are primarily synthesized *via* the phenylalanine pathway in leguminous plants, and are especially abundant in soybean seeds (Aoki et al., 2000). These metabolites not only play important anti-pathogenic roles in plants (Dixon et al., 2002; Subramanian et al., 2005; Graham et al., 2007; Meng et al., 2011; Zhang et al., 2020), and affect plant resistance to various abiotic stressors (Caldwell et al., 2005; Wu et al., 2008; Gutierrez-Gonzalez et al., 2010; Hamayun et al., 2017), but also benefit human health by reducing the risk of several diseases (Bradbury et al., 2014; Spagnuolo et al., 2015; Malloy et al., 2018). For example, previous studies have showed that soybean isoflavones play an important role in resistance to *Phytophthora sojae* (*P. sojae*) (Subramanian et al., 2005; Graham et al., 2007). Long term water deficit condition limited the isoflavone accumulation in soybean seeds (Gutierrez-Gonzalez et al., 2010). Isoflavone contents significantly increased in leaves and seeds of soybean under salt induction and over-expression of genes involved in isoflavone accumulation could improve soybean tolerance to salt (Jia et al., 2017).

In soybean seeds, isoflavones contain 12 components: daidzein, genistein, glycitein, daidzin, genistin, glycitin, 6-o-acetyldaidzin, 6-o-acetylgenistin, 6-o-acetylglycitin, 6-o-malonyldaidzin, 6-o-malonylgenistin, and 6-o-malonylglycitin; these components are classed as aglycones, glycosides, acetylglycosides, or malonylglycosides (Funaki et al., 2015; Sugiyama et al., 2017). Malonylglycosides are typically the most abundant type of isoflavone in soybean seeds, while aglycones are the least (Sugiyama et al., 2017). Despite their relatively low abundance, aglycone isoflavones (primarily daidzein, genistein, and glycitein) are important in the human body due to their comparatively high phytoestrogen activity and bioavailability (Nielsen and Williamson, 2007). Thus, it is critical to modify isoflavone quantity and composition to increase the relative abundance of aglycone isoflavones in soybean seeds, in order to improve the nutritional qualities of soy-derived foods.

Soybean isoflavone content is greatly influenced by both genetic and environmental factors during seed development (Zhang et al., 2014; Pei et al., 2018; Azam et al., 2020). The most influential environmental factors are climate, planting location, and year-to-year differences; of these, differences among years are the most important (Hoeck et al., 2000; Lee et al., 2003; Zhang et al., 2014). Recently, Azam et al. (2020) used 2 years of data collected for 1168 soybean accessions from three locations in the major ecoregions of soybean production in China to show that isoflavone content differed significantly among soybean accessions, accession types, growth years, and growth ecoregions.

As a typical quantitative trait, isoflavone content is controlled by multiple major and minor genes or quantitative trait loci (QTL); these QTLs are strongly affected by environmental and genetic factors (Primomo et al., 2005; Gutierrez-Gonzalez et al., 2011; Azam et al., 2020). To date, more than 200 QTLs for isoflavones distributed across the 20 soybean chromosomes are available in the SoyBase databank^1^. For example, Li et al. (2014) used a high-density genetic map that included 9948 polymorphic markers to identify 11 QTLs associated with isoflavone concentrations; Akond et al. (2014) identified three QTLs for soybean seed isoflavones using recombinant inbred line (RIL) populations and 5376 single nucleotide polymorphisms (SNPs) from the SoySNP6K BeadChip array (Illumina); and Cai et al. (2018) fine-mapped 15 stable QTLs for both individual and total isoflavone content using a high-density genetic linkage map with 3469 recombinant bin markers. However, with the exception of these few QTLs, most of the available isoflavones-related QTLs were obtained using lower-density genetic maps and do not overlap well. Because marker-assisted selection (MAS), which is based on QTLs, is a useful method by which to cultivate soybean varieties with high or low isoflavone concentrations (Akond et al., 2014), additional high-density genetic maps, containing abundant markers that cover the whole soybean genome, are still needed to identify the QTLs or genes most highly associated with isoflavone concentrations.

Therefore, in this study, we aimed to identify and fine-map a locus associated with soybean isoflavone concentration using a high-density genetic map with 2647 bin makers constructed by 119 RILs. We then aimed to isolate the candidate gene at this locus, and verify its association with isoflavone concentration using genetic transformation assays. Finally, we aimed to preliminarily investigate the roles of the candidate gene in response to abiotic and biotic stressors, including salt, drought, and pathogen infection.

## Materials and Methods

### Mapping Population Construction

Two soybean accessions, “Zhongdou27” with high isoflavone content and “Dongong8004” with low isoflavone content, were crossed to develop RILs, using the single seed descent (SSD) method (Oldach et al., 2014). An expanded population with 119 F_5:18_ families was derived and used to construct a linkage map and detect the QTLs associated with isoflavone content.

For fine-mapping of the identified QTL (qISO19-1), a BC_2_F_3_ population of 500 lines was constructed by backcrossing “Zhongdou27” (donor parent) and “Dongnong8004” (receptor parent). Heterozygosity at qISO19-1 was detected using recurrent selection in the BC_2_F_2_ population, and isoflavone contents were measured in the BC_2_F_3_ seeds produced by selected BC_2_F_2_ plants.

### Field Experiments

In preliminary experiments, “Zhongdou27” (male parent) and “Dongnong8004” (female parent) were planted in chernozem soils in Xiangyang (45°72′ N, 126°68′ E) and Hulan (45°9′ N, 126°58′ E) to confirm differences in isoflavone content. Subsequently, the RIL population was planted in Xiangyang and Hulan in 2018. For fine mapping, the BC_2_F_3_ population (as a transient generation) was planted in Xiangyang only (latitude 45°80′ N, 126°53′ E) in 2019. For candidate identification, a set of soybean germplasms consisting of 43 accessions were planted at Xiangyang in 2019 (Supplementary Table 1). Field trials were conducted in single-row plots (between 3 m long and 0.65 m rows) using a randomized complete block design, with three replicates per tested environment. Field management practices were typical, and were identical across environments. From all soybean materials, 20 seeds were collected from three plants per plot for isoflavone content measurement at the R8 stage (full maturity).

### Evaluation of Soybean Seed Isoflavone Content

Isoflavone contents in the seeds collected from the parental lines, the RILs, the BC_2_F_3_ population, and 43 soybean germplasms were measured using high-performance liquid chromatography (HPLC) as described previously (Vyn et al., 2002; Zeng et al., 2009). Briefly, 20 seeds from each soybean line were ground into a powder. Isoflavones were extracted by adding 0.1 g of this powder to 10 mL of 80% (v/v) ethanol in a 15 mL falcon tube. The mixture was slowly vortexed for 1 h and then left overnight. Next, the extraction mixture was hydrolyzed using 2 mL HCl solvent (2 mol/L). The mixture was filtered through a nylon membrane filter (0.22 μm; Thermo Fisher Scientific, United States), and isoflavone content was measured in 1.5 μl of the filtrate using an HPLC (1290 Infinity II; Agilent, United States) with reversed-phase HPLC columns (ZORBAX SB-C18, Agilent, United States; 4.6 mm × 250 mm; 5 μm). Solvent A was double-distilled water (ddH_2_O), and solvent B was methanol (chromatographic purity). The ratio of solvent A to solvent B ratio was 1:1, the solvent flow rate was 0.8 mL/min, and the temperature of the column was maintained at 50°C. Using an Agilent 1290 DAD detector, UV spectra were measured at 254 nm, and area responses were integrated using Agilent OpenLAB Control Panel software. The three major isoflavone components (daidzein, genistein, and glycitein) were identified and quantified based on standards purchased from the Chengdu Manster Biotechnology Co., Ltd. (China). Total isoflavone content was equivalent to the sum of the daidzein, genistein, and glycitein contents.

### Genotyping and Linkage Map Construction

Genomic DNA for the RIL population and the parental lines were prepared as described By Qi et al. (2014). Sequencing libraries for these samples were constructed and sequenced on an Illumina HiSeq2500 sequencing platform, following the manufacturer’s instructions. The sequencing reads for the RIL population and the parental lines were aligned to the soybean reference genome (assembly Glycine_max_v2.1) (Schmutz et al., 2010) using Short Oligonucleotide Alignment Program 2 (SOAP2) (Li et al., 2009). GATK (McKenna et al., 2010) was used to identify polymorphic SNPs between the RIL population and the parental lines.

Co-segregating SNPs were separated into bins, and a bin map was constructed based on the recombinant breakpoints of the RIL population with HighMap (Liu et al., 2014). Genetic distances among markers were calculated using the Kosambi mapping function (Kosambi, 1944). Linkage groups were discriminated at a log-likelihood threshold of 3.0. QTL mapping were performed using IciMapping v4.1 (Meng et al., 2015). Putative QTLs were identified based on LOD threshold of 2.5.

### Fine Mapping of qISO19-1

We used 500 lines in the BC_2_F_3_ family to construct a local saturation map within qISO19-1 based on SSR markers. The SSR primers used for fine-mapping are given in Supplementary Table 2. We measured isoflavone contents in the seeds produced by these lines as described above. Recombinants were identified in the fine-mapping population based on 12 polymorphic SSR markers, and the QTLs significantly associated with isoflavone content (LOD > 2.5) were identified using IciMapping v4.1 (Meng et al., 2015). Student’s *t*-test was used to identify significant differences between lines of BC_2_F_3_ with high and low content of isoflavone in soybean seed.

### Quantitative Real-Time PCR

For candidate gene identification, seeds of the 43 soybean germplasms described above were sampled at the R7 stage (yellow ripening stage); three replicate seeds were collected per accession for RNA extraction and isoflavone content measurement, to determine the correlation between isoflavone content and transcript abundance.

To analyze the relative expression dynamics of *GmMT1* between high- and low- isoflavone soybean cultivars, developing seeds of “Zhongdou27” and “Dongnong8004” were sampled every 7 days from developmental stage R5 to R8 (three replicate seeds per cultivar were sampled at every time point). Plants were grown in a greenhouse under a 16 h light/8 h dark photoperiod at 25–26°C until sampling.

To determine the involvement of *GmMT1* in the stress response, “Zhongdou27” was exposed to drought and salt stress. First, the roots of 3 week old “Zhongdou27” seedlings were immersed in quarter-strength (1/4) Murashige and Skoog (MS) liquid medium. We then supplemented the MS media of five seedlings with 150 mM NaCl, and the MS media of five seedlings with 8% (w/v) PEG6000 for the salt- and drought-stress response tests, respectively. The remaining five seedlings were kept in unsupplemented MS media as controls. Half of the uppermost fully extended leaf per seedling was sampled at 0, 1, 2, 4, 6, 8, 12, and 24 h after supplementation. During this period, plants were maintained in a greenhouse under a 16 h light/8 h dark photoperiod at 25–26°C. Harvested leaves were immediately frozen in liquid nitrogen and stored at −80°C. Leaves from three of the five treated plants were used for qRT-PCR.

qRT-PCRs were performed to determine the transcript abundance of *GmMT1* in soybean seeds or leaves. Total RNA was isolated from leaves or seeds using RNAprep pure Plant Kits (DP432, Tiangen). First-strand cDNA was synthesized from total RNA using TIANScript RT Kits (KR104, Tiangen). qRT-PCRs were performed on an ABI 7500 Fast platform using SuperReal PreMix Plus (SYBR Green) Kits (FP205, Tiangen). Each qRT-PCR (20 μl) included 2 μl cDNA, 10 μl 2 × SuperReal PreMix Plus, 0.4 μl 50 × ROX Reference Dye^Δ^, 5 μl of each forward and reverse primer, and 6.6 μl ddH_2_O_2_. The qRT-PCR amplification conditions were 95°C for 2 min, followed by 40 cycles of 95°C for 10 s, 60°C for 30 s, and 72°C for 30 s. The primers used are given in Supplementary Table 3. Three technical replicates were performed per sample, and the relative levels of transcript abundance were calculated using the 2^–ΔΔCT^ method (Livak and Schmittgen, 2001). The housekeeping gene *GmActin4* (GenBank accession no. AF049106) was used as the internal standard. Student’s *t*-test was used to identify significant differences in transcriptional abundance of *GmMT1*.

### *GmMT1* Cloning, Sequence Analysis, and Vector Construction

We predicted the 3-D structure of the putatively encoded protein GmMT1 using Phyre 2 (Kelley et al., 2015). We identified DNA and protein sequences homologous to *GmMT1* in soybeans and 15 other plant species in the Phytozome database^2^. We aligned these methyltransferases using DNAMAN (version 7.212, Lynnon Corp., QC, Canada). We then constructed a phylogenetic tree based on this alignment in MEGA 5 (Tamura et al., 2011).

The full-length cDNA sequence of *GmMT1* was amplified from the developing seeds of the high-isoflavone cultivar “Zhongdou27” using RT-PCR. RT-PCRs were performed using the KOD One PCR Master Mix (Code No. KMM-201; Toyobo (Shanghai) Biotech Co., Ltd., China), following the manufacturer’s instructions. The primers used were *GmMT1*-F and *GmMT1*-R, which were designed based on sequences flanking *GmMT1* in the Phytozome database (Goodstein et al., 2012), with the 5′ ends modified to include *Bgl*II and *Bst*EII restriction sites (Supplementary Table 3). The RT-PCR cycling conditions were as follows: 5 min at 94°C; 35 cycles of 30 s at 94°C, 30 s at 60°C, and 45 s at 72°C; and a final 10 min at 72°C. The purified PCR products were ligated into the pGM-T vector (VK207, Tiangen). Positive clones expressing the correct sequence were further inserted into pCAMBIA3301 vector using double digestion and ligation. The *bar* gene was used as a selection marker in the pCAMBIA3301 vector. Two expression vectors (35S:*GmMT1* or 35S:*bar*) were constructed for transformation using the recombinant pCAMBIA3301 plasmid.

### Subcellular Localization of *GmMT1*

The full-length coding region of *GmMT1* was inserted into the pCAMBIA1302 vector under the control of the 35S promoter to generate a *GFP*-fused *GmMT1* vector (*35S:GmMT1-GFP*). This recombinant vector and the control vector (*35S:GFP*) were transfected into separate groups of *Arabidopsis* protoplasts following Yoo et al. (2007). The transfected cells were examined and imaged under a confocal laser scanning microscope (DMi8, Leica, China). The primer sequences *GmMT1-GFP-F* and *GmMT1-GFP-R* were used for subcellular localization (Supplementary Table 3).

### Plant Transformation

To verify that *GmMT1* expression was associated with isoflavone content in soybeans, we overexpressed this gene in *Arabidopsis* and soybean plants. We selected “Dongnong50,” a low-isoflavone soybean cultivar, and *A. thaliana* Col-0 as the recipients of genetic transformation. The *A. thaliana* Col-0 mutant SALK_012168C was obtained from the *Arabidopsis* Biological Resource Center (ABRC). Transgenic soybean plants were grown in a greenhouse under a 16 h light/8 h dark photoperiod at 25–26°C; T0–T3 transgenic *Arabidopsis* plants were grown in a growth chamber with a photoperiod cycle of 16 h light/8 h dark at 22°C.

To develop transgenic *Arabidopsis*, the *35S:GmMT1* construct was transferred into *Agrobacterium tumefaciens* EHA105, and then transformed into two *Arabidopsis* strains (the Col-0 ecotype and the *mt1* mutant) using on the floral dip method (Clough and Bent, 1998). Transgenic *Arabidopsis* lines were selected using phosphinothricin; *bar* and *GmMT1* gene expression in the selected plants was verified by PCR amplification using specific primers (3301-*bar*-F/R and 3301-35S-*GmMT1*-F/R; Supplementary Table 3). We generated three independent T2 transgenic lines per strain. Isoflavone concentrations in the T2 transgenic plants were measured using HPLC as described above. T2 transgenic plants with no separation were used in subsequent experiments.

To test whether *GmMT1* overexpression increased isoflavone production in the roots of low-isoflavone soybean cultivars, the *35S:GmMT1* recombinant plasmid was transformed into *Agrobacterium rhizogenes* strain K599, and transformed in to the hairy roots of cultivar Donong50 as previously described (Cao et al., 2009). After 2 weeks of cultivation, once the hairy roots had appeared, we removed ∼1 cm sections from the root tips of 205 plants. Using PCR and specific primers (3301-*bar*-F/R and 3301-35S-*GmMT1*-F/R; Supplementary Table 3), we confirmed *bar* and *GmMT1* gene expression in roots. Isoflavone concentrations in the transgenic roots were measured as described above.

To determine whether the expression of *GmMT1* influenced isoflavone content in soybean seeds, we then developed transgenic soybeans by introducing the recombinant plasmid into *Agrobacterium tumefaciens* strain EHA105, and performing stable transformation into the cotyledon nodes of soybean cultivar “Dongnong50” following Paz et al. (2004). The expression of *GmMT1* in leaves of T2 transgenic soybean plants was verified using PCR amplification (3301-*bar*-F/R and 3301-35S-*GmMT1*-F/R) (Supplementary Table 3), western blotting, and qRT-PCR. Isoflavone concentrations in seeds of the T2 transgenic plants were measured as described above. T2 plants expressing *GmMT1* were used for all subsequent experiments.

### Effects of Stress on Transgenic Plants

We tested the effects of salt and drought stress on the transgenic *Arabidopsis* and soybean plants. To test the effects of salt and drought on *Arabidopsis*, we planted wild-type (Col-0), *mt1* mutant, and T3 transgenic (*GmMT1-ox* and *GmMT1-ox mt1*) *Arabidopsis* on MS agar plates (five plates per strain). We then treated three plates per strain with 100 Mm NaCl, and three plates per strain with 300 mM mannitol. The remaining untreated plates were used as controls. All plants were kept at 4°C for 3 days in the dark, and then transferred to a 22°C environment with a photoperiod cycle of 16 h light/8 h dark. We calculated the germination rate on the 4th day after treatment and measured the root growth of all plants using a Vernier caliper.

To explore whether isoflavone accumulation, driven by *GmMT1* overexpression, improved soybean resistance to *P. sojae*, we measured *GmMT1* transcript abundance in transgenic and wild-type hairy roots, as well as the reaction of transgenic and wild-type hairy roots to *P. sojae* infection. Before *P. sojae* infection, we removed ∼1 sections from the hairy root tips of wild-type and *GmMT1*-overexpressing “Donong50” that had been cultured for 15 days. The relative expression levels of *GmMT1* were detected in these samples. Then, to test the effects of *P. sojae* infection, well-propagated cultures of *P. sojae* were cut into small pieces and placed on new carrot agar (CA) plates. The hairy roots were spread on the CA medium with the mycelia, and were cultured in a growth chamber under a 16 h light/8 h dark cycle at 25°C. After ∼14 days of incubation, once the hairy roots had appeared, we imaged the hairy roots to check for signs of infection.

## Results

### Phenotypic Evaluation of the Recombinant Inbred Line Populations

Two soybean varieties (*Glycine max* (L.) Merr), one with high isoflavone content (“Zhongdou27”), and one with low isoflavone content (“Dongnong8004”), were planted in two locations *GmMT1* Effects Soybean Isoflavone Content (Xiangyang and Hulan, China) to verify the difference in isoflavone content between the accessions. In both locations, isoflavone content was significantly higher in “Zhongdou27” than in “Dongnong8004” (*P* < 0.001) (Supplementary Table 4). Thus, these accessions were suitable for map-based QTL analysis. We therefore crossed “Zhongdou27” (male parent) with “Dongnong8004” (female parent) to derive an RIL mapping population of 119 F_5:18_ families. When the RIL were planted in Xiangyang and Hulan, isoflavone contents varied widely with location and population. Across the RIL population, total isoflavone content ranged from 1211.99 μg/g to 5683.43 μg/g (Supplementary Table 4). All traits of interest (i.e., total isoflavone content and content of each individual isoflavone) were continuously distributed (Supplementary Figure 1).

### Quantitative Trait Loci Mapping for Soybean Isoflavone Content

Genome resequencing was conducted to genotype the parental lines (“Zhongdou27” and “Dongnong8004”) and the 119 RILs in the mapping population. For “Zhongdou27,” we generated 26.18 GB of raw data, and 78.88% of the reads were successfully aligned to the soybean reference genome with an average depth of 24.54-fold; for “Dongnong8004,” we generated 25.58 GB of raw data, and 78.30% of the reads were successfully aligned to the soybean reference genome with an average depth of 22.86-fold (Supplementary Table 5). A total of 343,907 high-quality SNPs were identified between the two parents. Across all RILs, we generated 441.7 GB of raw data, with an average sequencing depth of 3.65-fold. For each of the 119 RILs, we generated an average of 3.46 GB of raw data (Supplementary Table 5). A total of 353 million SNPs were identified among the 119 RILs; all SNP sites in the RILs were integrated as recombination bin units. Finally, a genetic linkage map with 4231 bin markers was constructed along the 20 chromosomes (Supplementary Table 6). The total length of the bin map was 2172.98 centimorgans (cM), with a mean interval between markers of 0.82 cM (Supplementary Figure 2 and Supplementary Table 6).

**FIGURE 1:**
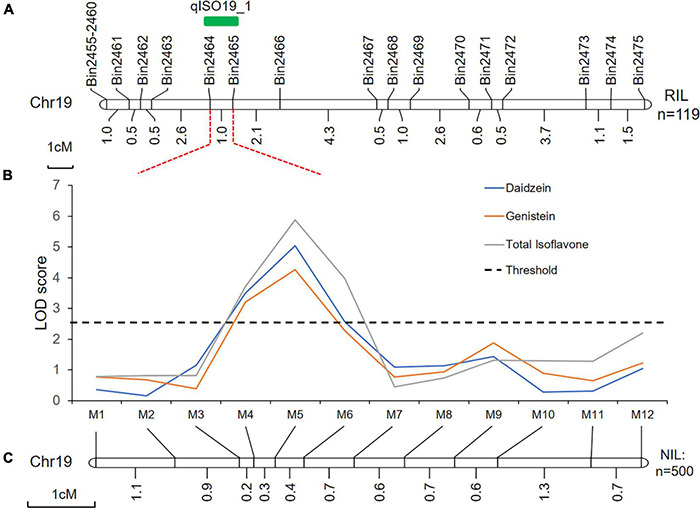
Genetic and physical maps of the qISO19-1 region. **(A)** Fine-mapping of the QTL qISO19-1 on soybean chromosome 19 using the RIL population. Genetic distances, in cM, are shown below the chromosome, and the locations of the markers and qISO19-1 are shown above. **(B)** LOD scores for isoflavone contents (daidzein and total isoflavone) over QTL qISO19-1 on chromosome 19. LOD scores were calculated independently by ICIM. The threshold LOD value was 2.5. **(C)** Genetic map showing the redefined position of qISO19-1 on chromosome 19 based on the BC_2_F_3_ population.

Using linkage mapping, we identified 15 QTLs associated with daidzein (DZ), genistein (GC), glycitein (GT), and total isoflavone contents (TI), covering 10 of the 20 soybean chromosomes. Almost all QTLs were detected in both locations (Xiangyang and Hulan) or as multiple-effect QTLs, controlling the abundance of two or more isoflavones; the one exception was QTL qGC1-2, which was only associated with glycitein content in seeds from plants grown in Xiangyang (Table 1 and Supplementary Figure 3). Four of the 15 QTLs have been previously reported (Table 1). The remaining 11 QTLs were novel (Table 1). One novel locus, qISO19-1, which was detected between markers Bin2464 and Bin2465 on chromosome 19, was identified both by the inclusive composite interval mapping method (ICIM) (Figure 1A and Table 1). This QTL controlled the contents of daidzein, genistein, and total isoflavone (Figure 1B and Table 1). The genetic contribution of this QTL to each of these traits was more than 10%, indicating that qISO19-1 was an important QTL controlling individual and total content of soybean isoflavone. Thus, we selected qISO19-1 for further study.

**TABLE 1:** Quantitative trait locis underlying soybean isoflavone content in two environments.

Chromosome	QTL	Left marker	Right marker	Position	Trait^a^	LOD	R^2^ (%)	Add	Novel or previously reported QTL
01	qGC1-1	Bin2	Bin3	90	GC_e1	2.89	7.92	−92.502	Novel
					GC_e2	3.04	8.23	−124.663	
01	qGC1-2	Bin89	Bin90	38	GC_e2	3.25	10.21	−93.461	Novel
02	qISO2-1	Bin156	Bin157		DZ_e1	3.18	9.16	−238.4209	Novel
					DZ_e2	4.06	11.02	−162.3418	
					GT_e1	3.58	9.23	−147.9449	
					TI_e1	4.51	12.39	−334.3811	
					TI_e2	3.76	10.97	−269.3571	
03	qTI3-1	Bin324	Bin325	5	TI_e1	3.45	11.09	301.1243	Seed isoflavone 4-2 (Liang et al., 2010)
					TI_e2	4.06	13.28	124.4489	
05	qISO5-1	Bin713	Bin717	99	DZ_e1	2.79	9.29	−225.5618	Novel
					DZ_e2	3.52	11.08	−162.4358	
					TI_e2	4.5	13.28	−220.8508	
08	qISO8-1	Bin1088	Bin1092	68	DZ_e1	2.65	9.08	−119.1458	Novel
					GT_e1	4.21	12.09	−150.1215	
					GT_e2	3.68	10.21	−153.7711	
					TI_e1	3.96	11.29	−331.0842	
08	qTI8-1	Bin1126	Bin1128	23	TI_e1	2.89	8.19	−123.1042	Seed total isoflavone 9-1 (Wang et al., 2015)
					TI_e2	4.82	12.09	−260.4606	
08	qISO8-2	Bin1140	Bin1141	5	DZ_e1	2.79	8.92	−123.6876	Novel
					DZ_e2	3.02	9.08	−126.9897	
					GT_e2	2.57	7.89	146.395	
					TI_e1	3.54	10.29	−307.761	
					TI_e2	5.63	19.27	−340.2377	
10	qISO10-1	Bin1383	Bin1384	87	DZ_e1	3.02	9.74	213.2513	Novel
					DZ_e2	2.59	7.95	157.2833	
					TI_e1	3.17	10.09	138.9982	
13	qISO13-1	Bin1733	Bin1734	93	DZ_e1	2.54	8.65	175.3847	qIF13-1(Cai et al., 2018)
					DZ_e2	3.07	9.34	163.4374	
					TI_e1	2.89	8.15	221.7404	
					TI_e2	3.28	10.79	356.7454	
13	qISO13-2	Bin1839	Bin1840	41	DZ_e1	3.59	10.14	−131.2053	Novel
					DZ_e2	3.87	9.87	−126.7739	
					GT_e2	2.55	8.74	−130.2816	
					TI_e2	3.41	11.02	−332.4529	
14	qISO14-1	Bin1964	Bin1967	35	DZ_e2	3.54	10.09	−177.8066	Novel
					GT_e1	2.78	9.08	−162.1882	
					GT_e2	3.05	9.76	−117.5672	
					TI_e2	2.57	8.59	−113.9187	
19	qISO19-1	Bin2464	Bin2465	78	DZ_e1	2.87	10.96	−122.8813	Novel
					DZ_e2	2.65	10.29	−129.4217	
					GT_e1	3.04	12.19	−144.6579	
					TI_e1	4.05	14.48	−307.761	
19	qISO19-2	Bin2470	Bin2471	67	DZ_e1	3.41	13.04	−128.4985	Novel
					TI_e1	3.06	11.49	−110.1879	
20	qISO20-1	Bin2581	Bin2586	43	DZ_e1	2.97	10.29	−178.9113	Novel
					DZ_e2	3.54	14.87	−180.2642	
					TI_e1	3.02	12.09	−238.4209	

*^a^e1 and e2 indicate two experimental locations “Xiangyang” and “Hulan,” respectively.*

### Fine Mapping of qISO19-1 and Candidate Gene Mining

**FIGURE 2:**
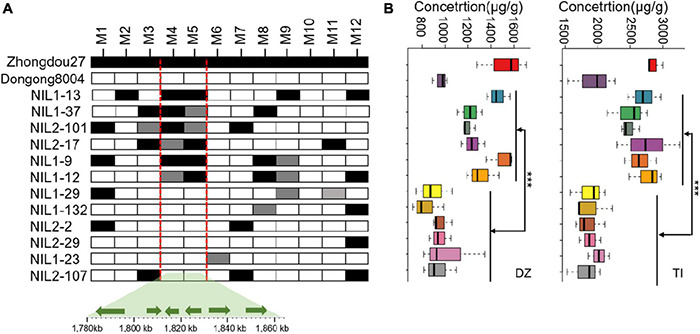
Refinement of the qISO19-1 region. **(A)** Restriction of qISO19-1 to a 62.8-kb region of chromosome 19 in the RIL population, and identification of recombinants based on 12 polymorphic markers. Black cells indicate alleles homozygous with the male parent (“Zhongdou27”); white cells indicate alleles homozygous with the female parent (“Dongnong8004”); gray cells indicate heterozygous alleles. Below are shown the positions of the six candidate genes in the qISO19-1 region, as indicated by comparisons to the reference genome. **(B)** Contents of daidzein (DZ) and total isoflavone (TI) in seeds produced by the recombinant lines. ***P < 0.001.

Quantitative trait loci qISO19-1 was fine-mapped using 75 simple sequence repeat (SSR) markers near qISO19-1 (between markers Bin2464 and Bin2465; Figure 1A). Of these SSR markers, 12 (M1–M12) were polymorphic between the parental lines, as well as within the BC_2_F_3_ population. The local saturation map of qISO19-1 for daidzein and total isoflavone contents showed that the logarithm of the odds (LOD) scores were above the threshold from M4 (SSR_19_0116) to M5 (SSR_19_0123) (Figures 1B,C). Within the fine-mapped population, we identified five recombinants that were homozygous for “Dongong8004” alleles at M4 and M5, and five recombinants that were homozygous or heterozygous for the “Zhongdou27” at these alleles (Figure 2A). Levels of daidzein and total isoflavone in the seeds produced by the lines carrying the “Zhongdou27” alleles were significantly higher (*P* < 0.001) than those produced by the lines carrying the “Dongong8004” alleles (Figure 2B). Thus, the qISO19-1 QTL was narrowly defined to a 62.9-kb region between markers SSR_19_0116 and SSR_19_0123 on chromosome 19. Based on comparisons with the reference genome (*G. max* Williams 82) (Schmutz et al., 2010), this interval harbors five putative genes (Figure 1A): *Glyma.19G017200, Glyma.19G017300, Glyma.19G017400, Glyma.19G017500*, and *Glyma.19G017700* (Supplementary Table 7).

Relative expression levels of these five genes were quantified for 43 soybean germplasms: 20 germplasms with high isoflavone content (3709—5970 μg/g) and 23 germplasms with low isoflavone content (1263—2042 μg/g) (Supplementary Table 1). Four genes, *Glyma.19G017200, Glyma.19G017300, Glyma.19G017400*, and *Glyma.19G017500*, were expressed in the seeds of all accessions during the late R6 stage (Supplementary Figure 4). *Glyma.19G017700* was expressed at a relatively low level in 30 accessions (Supplementary Figure 4). The relative expression level of *Glyma.19G017500* was significantly positively correlated with isoflavone content in the seeds of all accessions (Supplementary Table 8). Thus, we considered *Glyma.19G017500*, which is a methyltransferase (MT), a candidate gene at the qISO19-1 locus. This gene was named *GmMT1*.

### *GmMT1* Sequence Analysis and Localization

The full-length coding sequence (CDS) of *GmMT1* (*Glyma.19G017500*) from the high-isoflavone cultivar “Zhongdou27” was 957 bp long; this sequence was 99.16% consistent with the reference genome (*G. max* Williams 82; Schmutz et al., 2010; Supplementary Figure 5a). *GmMT1* encoded a putative protein composed of 318 amino acid with a predicted molecular mass of 35.16 kDa. This protein harbored all 20 amino acids, with the most abundant being leucine and least abundant being tryptophan. The calculated instability and aliphatic indexes of this protein were 51.94 and 89.47, respectively, suggesting that the protein was unstable. The predicted 3-D structure suggested that *GmMT1* encoded an m^2^ G966 specific 16S rRNA methyltransferase (Lesnyak et al., 2007; Supplementary Figure 5b). Using the Phytozome database (Goodstein et al., 2012), we identified a second copy of *GmMT1* on chromosome 13 (*Glyma.13G066900*); the amino acid sequence putatively encoded by this copy was 82.15% homologous with *GmMT1* (Supplementary Figure 6). Phylogenetic analysis of GmMT1 and several other plant methyltransferases indicated that GmMT1 formed a well-supported clade with the methyltransferases of *Phaseolus vulgaris*, *Medicago truncatula*, and *Trifolium pratense* (Supplementary Figure 5c), suggesting that the functions of *MT1* may be conserved across leguminous plants.

### Expression of *GmMT1* in Developing Soybean Seeds and in Response to Stress

**FIGURE 3:**
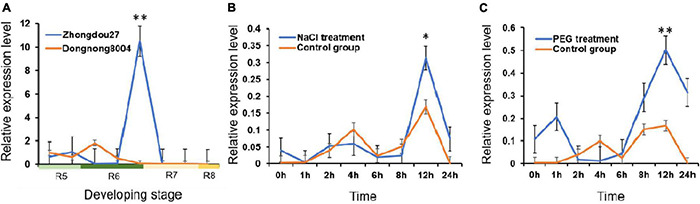
The expression patterns of *GmMT1* in soybeans. **(A)** Relative expression of *GmMT1* in the seeds of the high-isoflavone cultivar “Zhongdou27” and low-isoflavone cultivar “Dongnong8004” during development. Seeds were sampled every 7 days from the start of stage R5 to the end of stage R8. **(B,C)** Expression of *GmMT1* in soybean leaves in response to panels **(B)** salt and **(C)** drought stress. Time represents the hours after stress initiation. *n* = 3 samples per stage or time point. “^**^” and “*” indicate *P* < 0.01 and *P* < 0.05 based on Student’s two-tailed *t*-test.

At developmental stage R7, *GmMT1* was significantly upregulated in the seeds of the high-isoflavone cultivar “Zhongdou27” as compared to the seeds of the low-isoflavone cultivar “Dongnong8004” (Figure 3A). At all other developmental stages tested, *GmMT1* expression level did not differ between cultivars. However, the stark difference in *GmMT1* expression at stage R7 suggested that this gene may participate in isoflavone accumulation in soybean seeds. *GmMT1* expression was also induced in the high-isoflavone cultivar “Zhongdou27” in response to salt and drought stress (Figures 3B,C). In both cases, *GmMT1* was significantly upregulated at 12 h after stress initiation in comparison to the control group (Figures 3B,C).

### The *GmMT1* Protein Was Expressed in the Chloroplast

**FIGURE 4:**
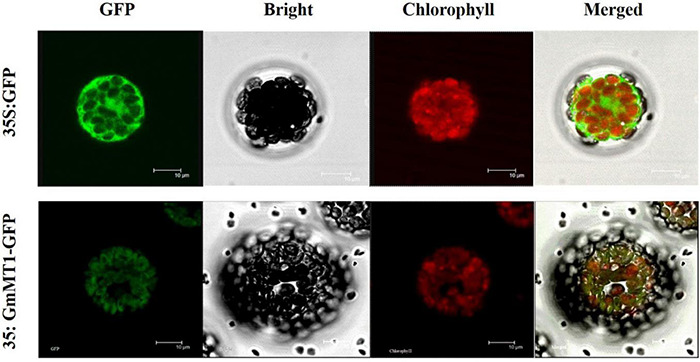
Sub-cellular localization of the GmMT1 protein in *Arabidopsis* protoplasts. GmMT1-GFP expression was driven by the cauliflower mosaic virus 35S promoter and transiently expressed in *Arabidopsis* protoplasts. The images shown are GFP fluorescence (green) only, bright-field, chlorophyll auto-fluorescence (red) only, and combined. Scale bars = 10 μm.

We expressed the green fluorescent protein (GFP) and the GmMT1-GFP fusion protein, both under the control of the 35S promoter, in separate *Arabidopsis* protoplasts. In protoplasts carrying 35S:GFP, GFP signal was dispersed throughout the cell (Figure 4). However, in protoplasts carrying 35S:GmMT1-GFP, the GFP signal was primarily observed in the chloroplasts (Figure 4), indicating that GmMT1 was a chloroplast-localized protein.

### Overexpression of *GmMT1* Increased Isoflavone Concentrations in Transgenic Plants

We used *Agrobacterium rhizogenes*-mediated transformation to overexpress *GmMT1* in the hairy roots of soybean cultivar “Donong50,” a low-isoflavone cultivar. In the hairy roots overexpressing *GmMT1* (Supplementary Figures 7a,b), total isoflavone concentrations were significantly greater than in the wild-type hairy roots (Supplementary Figure 7c).

**FIGURE 5:**
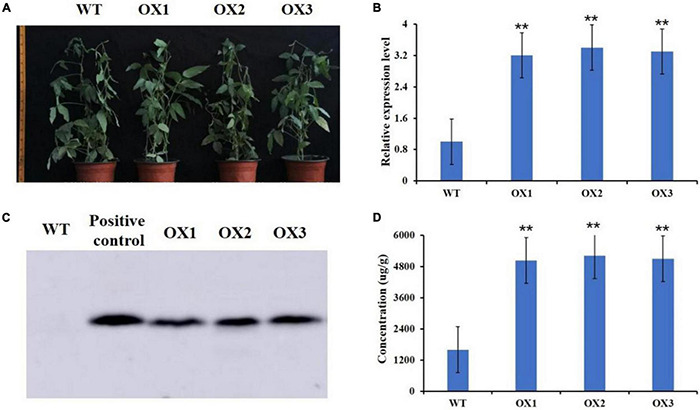
Overexpression *GmMT1* in the soybean cultivar “Dongnong50” (WT). **(A)** WT Dongnong plants and T2 transgenic plants overexpressing *GmMT1* (OX1–3). **(B)** Relative *GmMT1* expression in WT and T2 transgenic plants. **(C)** Western blot showing the expression of the bar protein in the transgenic soybean plants. **(D)** Isoflavone contents in the seeds produced by the WT and transgenic soybean cultivars. ** indicates a significant difference between WT and transgenic soybean plants (*P* < 0.01, Student’s *t*-test. Error bars represent the standard error, *n* = 3).

We then used *Agrobacterium tumefaciens*-mediated transformation to co-overexpress *GmMT1* and the selection marker gene *bar* in “Dongnong50,” a low-isoflavone cultivar that is also an excellent transgenic receptor, to generate three independent T2 transgenic soybean lines (Figure 5A). In the seeds of the T2 transgenic lines, *GmMT1* was significantly upregulated as compared to non-transgenic “Dongnong50” (Figure 5B), and western blots confirmed the expression of the bar protein in the transgenic plants (Figure 5C). Isoflavone contents in the seeds of the *GmMT1*-overexpressing T2 transgenic lines were significantly greater than isoflavone contents in the seeds of the wild-type plants (increases of nearly 3.0-fold; Figure 5D). Thus, our results suggested that *GmMT1* expression might be associated with isoflavone biosynthesis in both soybeans and *Arabidopsis*.

### *GmMT1* Overexpression Improved Arabidopsis Resistance to Salt and Drought

**FIGURE 6:**
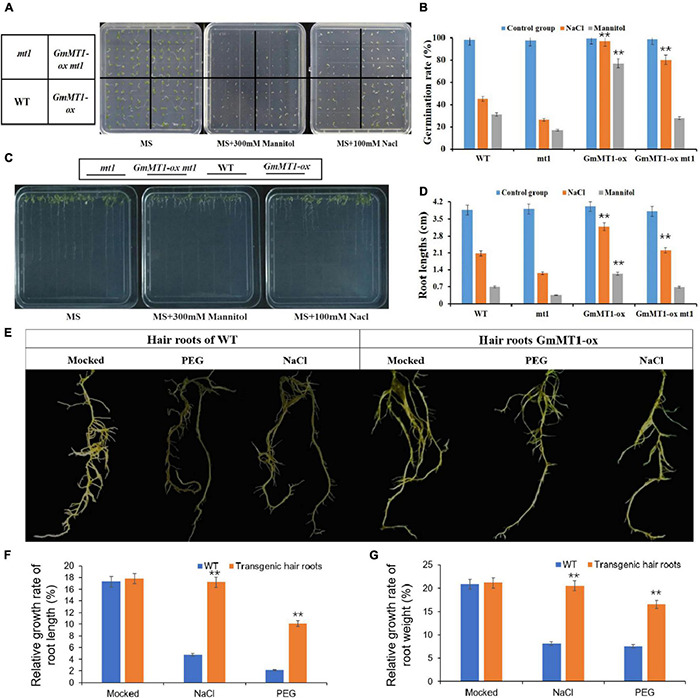
Effects of *GmMT1* overexpression on *Arabidopsis* and on soybean hairy roots after exposure to salt or drought stress. **(A,B)** Germination and **(C,D)** root growth of *Arabidopsis* Columbia-0 (WT), *Arabidopsis mt1* (a mutant strain in which the *Arabidopsis* homolog of*GmMT1*, *At3G28460*, is silenced), and two T3 transgenic lines overexpressing *GmMT1* (*GmMT1-ox* and *GmMT1-ox mt1*) after exposure to salt (100 mM NaCl) or drought (300 mM mannitol) stress. **(E)** Phenotypic differences in hairy roots of transgenic and non-transgenic soybeans after exposure to salt (NaCl) or drought (PEG) stress. **(F,G)** Relative rates of increase in panels **(F)** root length and **(G)** root weight for non-transgenic “Dongnong50” soybeans (WT) and transgenic soybeans overexpressing *GmMT1* after exposure to salt (NaCl) or drought (PEG) stress. ** indicates a significant difference between WT and transgenic or between treatment and control groups (*P* < 0.01, Student’s *t* test). Error bars represent standard error (*n* = 3).

Using *Agrobacterium*-mediated transformation, we overexpressed *GmMT1* in wild-type *Arabidopsis thaliana* Columbia-0 (Col-0) and in *A. thaliana mt1*, a mutant strain in which the *Arabidopsis* homolog of *GmMT1* (At3G28460) was silenced, to generate *GmMT1-ox* and of *GmMT1-ox mt1*; three independent T3 lines of each transgenic strain were generated (Supplementary Figures 8a–c). Under control conditions, germination rate did not differ among wild-type (Col-0), *mt1* mutant, and T3 transgenic (*GmMT1-ox* and *GmMT1-ox mt1*) *Arabidopsis* seeds (Figures 6A,B). When exposed to salt or drought stress, seed germination rate in the wild-type and the *mt1* mutant decreased significantly (Figures 6A,B). In the *GmMT1-ox* plants, seed germination rate decreased in response to both salt and drought stress (significantly in the latter case), but remained significantly greater than those in stressed non-*GmMT1*-overexpressing plants (Figures 6A,B). In contrast, in *GmMT1-ox mt1* plants, seed germination rates decreased significantly in response to both salt and drought stress, and seed germination was significantly greater than stressed non-*GmMT1*-overexpressing plants after salt stress, but not drought stress (Figures 6A,B). Root growth measurements returned similar results to the seed germination assays. That is, after salt or drought stress, the roots of transgenic wild-type and *mt1 Arabidopsis* plants were longer than those of plants not overexpressing *GmMT1* (Figures 6C,D).

The relative growth rates of the hairy roots of low-isoflavone soybean cultivar Donong50 decreased significantly with respect to both length and weight after exposure to drought or salt stress (Figures 6E–G). However, the hairy roots of transgenic Donong50 overexpressing *GmMT1* were significantly more tolerant of drought and salt stress (Figures 6E–G). Indeed, as compared to the non-transgenic cultivar, relative rates of root length and weight increase in the transgenic cultivar were respectively 12.13% and 11.78% greater after salt stress, and respectively 7.91% and 9.15% greater after drought stress (Figures 6E–G). Thus, *GmMT1*-driven increases in isoflavone concentrations might increase the resistance of plants to salt and drought stress.

### *GmMT1* Overexpression Improved Soybean Resistance to *Phytophthora sojae*

**FIGURE 7:**
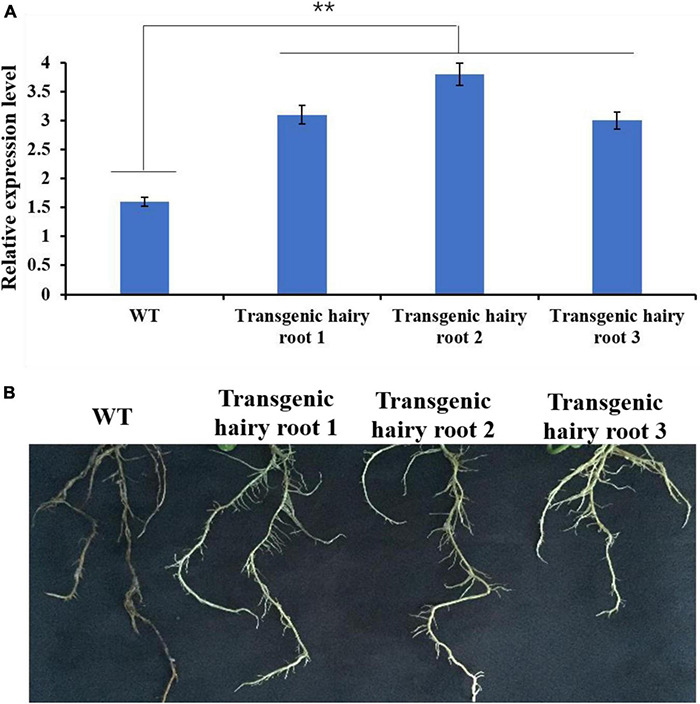
The effects of *GmMT1* overexpression on *Phytophthora sojae* resistance. **(A)** The relative expression levels of *GmMT1* in non-transgenic low-isoflavone soybean cultivar Donong50 (WT) and three transgenic Donong50 lines overexpressing *GmMT1.* ** indicates a significant difference between WT and transgenic hair roots (*P* < 0.01, Student’s *t*-test). **(B)** Phenotypic differences in hairy roots of transgenic and non-transgenic soybeans after *P. sojae* infection.

Under control (uninfected) conditions, *GmMT1* was significantly more highly expressed in the three transgenic Donong50 lines overexpressing *GmMT1* than in the non-transgenic low-isoflavone soybean cultivar Donong50 (Figure 7A). Correspondingly, the symptoms of *P. sojae* infection, including watery and rotting lesions, were less obvious in the transgenic hairy roots as compared to the non-transgenic hairy roots after 2 weeks of treatment (Figure 7B). Thus, our results indicated that the overexpression of *GmMT1* might increase the resistance of soybeans to *P. sojae* infection, and/or reduce the severity of the infection.

## Discussion

Quantitative trait loci mapping is the most common and effective method used to analyze agronomically important plant characteristics (Pulst, 1999; Rafalski, 2010). Soybean isoflavone content is a quantitative trait, controlled by multiple genes, which is easily affected by the environment (Zeng et al., 2009). Here, we identified 15 QTLs underlying soybean isoflavone content, of which three overlapped with previously reported QTLs in genomic regions. Of these three QTLs, qTI3-1 overlapped with the reported QTL “Seed isoflavone 4-2” associated with soybean isoflavone content (Liang et al., 2010); qTI8-1 occupied a similar genomic region to the reported QTL “Seed total isoflavone 9-1” (Wang et al., 2015); and qISO13-1 overlapped with the known QTL “qIF13-1” (Cai et al., 2018).

Using the 12 novel QTLs, we fine-mapped the important stable locus qIF19-1, and identified *GmMT1* as a candidate gene associated with isoflavone content at this locus.

*GmMT1* encodes an m^2^ G966-specific 16S rRNA methyltransferase, which falls into the S-adenosyl-L-methionine-dependent methyltransferase (SAM-Mtase) superfamily (Joshi and Chiang, 1998; Lesnyak et al., 2007; Zou et al., 2020). SAM-Mtases are key enzymes in many plant metabolic pathways, playing important roles in the biosynthesis of many plant products associated plant growth and development, as well as in the resistance to many biotic and abiotic stressors (Douglas, 1996; Ying et al., 1996; Song et al., 2009; Byeon et al., 2015; Nam et al., 2016; Choi et al., 2017; Niu et al., 2018; Zou et al., 2020). The m^2^ G966-specific 16S rRNA methyltransferases participate in specific methylation of the G966 base of 16S rRNA (Lesnyak et al., 2007). Recently, Zou et al. (2020) localized *CMAL*, a 16S rRNA methyltransferase, to the chloroplast, and showed that this protein was important for chloroplast ribosome biogenesis and plant development. However, few other studies of the biological functions of 16S rRNA methyltransferases are available.

Here, we fine-mapped and cloned a soybean methyltransferase gene, *GmMT1*, that was strongly associated with isoflavone accumulation in soybean seeds. Consistent with Zou et al. (2020), we localized GmMT1 to the chloroplast. Moreover, we found that the hairy roots of transgenic low-isoflavone soybean lines overexpressing *GmMT1* had significantly greater isoflavone contents than the hairy roots of the same lines not overexpressing *GmMT1*, suggesting that *GmMT1* may participate the isoflavone biosynthesis in soybeans. Additionally, we found that the heterologous expression of *GmMT1* in *Arabidopsis*, both ecotype Col-0 and the *mt1* mutant, increased isoflavone content relative to non-transgenic lines. This suggested that *GmMT1* may regulate isoflavone biosynthesis in other plants as well as soybeans.

Previous studies have shown that isoflavone content may affect plant resistance to various abiotic stressors, including salt and drought (Caldwell et al., 2005; Wu et al., 2008; Gutierrez-Gonzalez et al., 2010). In addition, decreases in isoflavone content have been shown to weaken the resistance of soybeans to the pathogen *P. sojae* (Subramanian et al., 2005; Graham et al., 2007). Here, we found that transgenic soybean and *Arabidopsis* lines overexpressing *GmMT1* not only contained higher levels of isoflavones than non-transgenic lines, but were less susceptible to salt and drought stress. This may be because isoflavones commonly exist as water-soluble glycosides (Pandey et al., 2014), which may play an important role in osmotic regulation under drought and salt stress (Pandey et al., 2014). In addition, when soybeans were infected with *P. sojae*, the hairy roots of the transgenic lines overexpressing *GmMT1* exhibited milder symptoms of *P. sojae* infection than did the non-transgenic lines, supporting the association between soybean isoflavone content and *P. sojae* resistance. Similarly, Cheng et al. (2015) found that an isoflavone reductase gene, *GmIFR*, was related to soybean isoflavone content and *P. sojae* resistance. However, the functional effects of *GmMT1* and *GmIFR* on soybean isoflavone content and *P. sojae* resistance differed. Unlike *GmMT1, GmIFR* expression decreased isoflavone content and increased *P. sojae* resistance.

Our results showed that *GmMT1* participates in the regulation of isoflavone content in soybeans, and that biotic and abiotic stress resistance depend on isoflavone content, and, consequently, *GmMT1* expression. However, further study of *GmMT1* is required to better characterize the regulatory mechanisms underlying isoflavone accumulation, as well as the effects of isoflavone content on the stress responses of soybeans and other plants.

## Data Availability Statement

The original contributions presented in the study are included in the article/Supplementary Material, further inquiries can be directed to the corresponding author/s.

## Author Contributions

XZ and YH designed and supervised the research. RL, JZ, and DS conducted the experiment and analyzed the data. DW, ML, YZ, and WT conducted the field trial. XZ, YJ, YH, and WL wrote the manuscript. All authors read and approved the manuscript.

## Conflict of Interest

The authors declare that the research was conducted in the absence of any commercial or financial relationships that could be construed as a potential conflict of interest.

## Publisher’s Note

All claims expressed in this article are solely those of the authors and do not necessarily represent those of their affiliated organizations, or those of the publisher, the editors and the reviewers. Any product that may be evaluated in this article, or claim that may be made by its manufacturer, is not guaranteed or endorsed by the publisher.

## Footnotes

1
www.soybase.org

2
https://phytozome.jgi.doe.gov/pz/portal.html
