# Supplementary material for: Fine-Mapping and Functional Analyses of a Candidate Gene Controlling Isoflavone Content in Soybeans Seed
Source: Front Plant Sci. 2022 Apr 25;13:865584. doi: 10.3389/fpls.2022.865584 (PMC9084227; doi:10.3389/fpls.2022.865584)
Supplement: Supplementary file 1 [file Data_Sheet_1.docx]

**
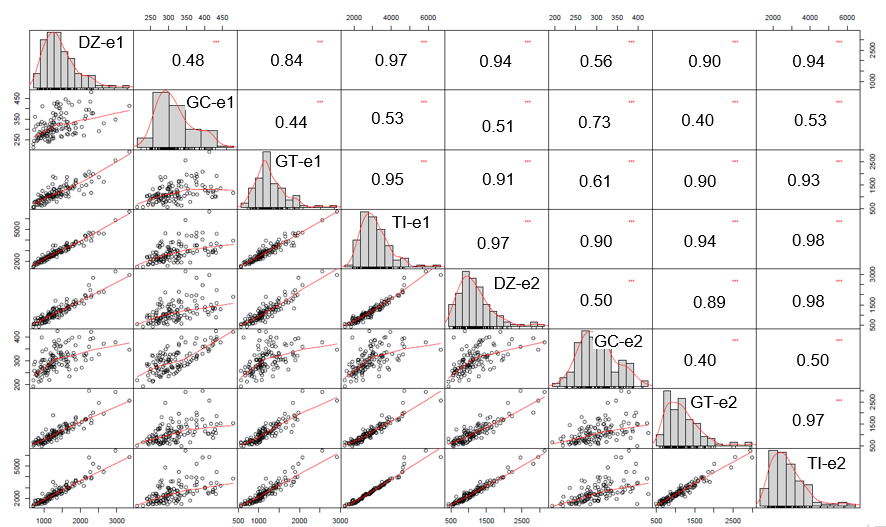
**

Figure S1 Soybean isoflavone contents in the RIL population.

The frequency distributions for DZ, GC, GT, and TI content are listed along the diagonal; the trendline of each histogram shows the normal curve. e1 and e2 represent the two experimental locations, ‘Xiangyang’ and ‘Hulan’, respectively. The pairwise correlations between these traits are shown above and below the diagonal.


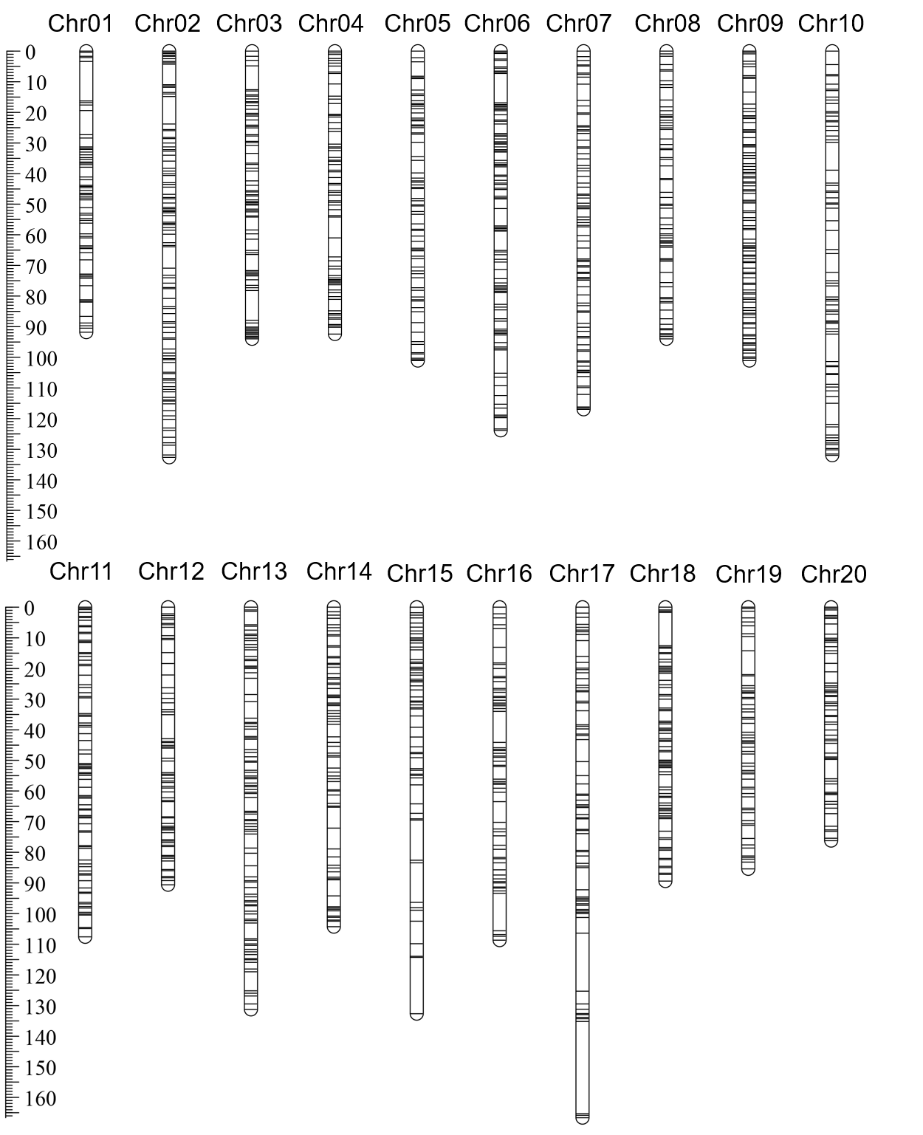


Figure S2 Genetic linkage map of the bin markers along the soybean chromosomes based on the SNPs identified among the 119 RILs.


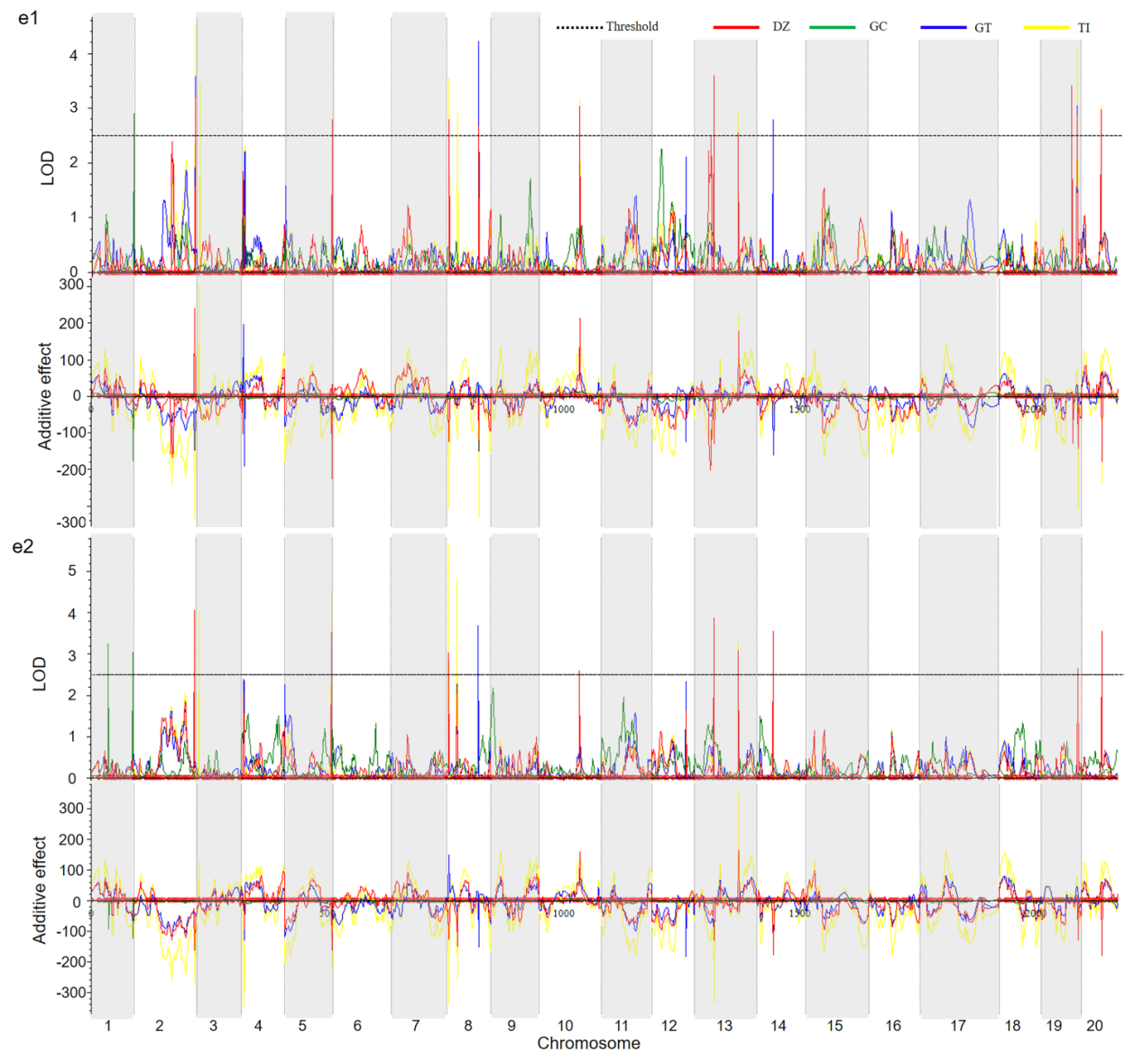


Figure S3 QTL mapping results of soybean isoflavone content in two environments.

e1 and e2 indicate two experimental locations ‘Xiangyang’ and ‘Hulan’, respectively.


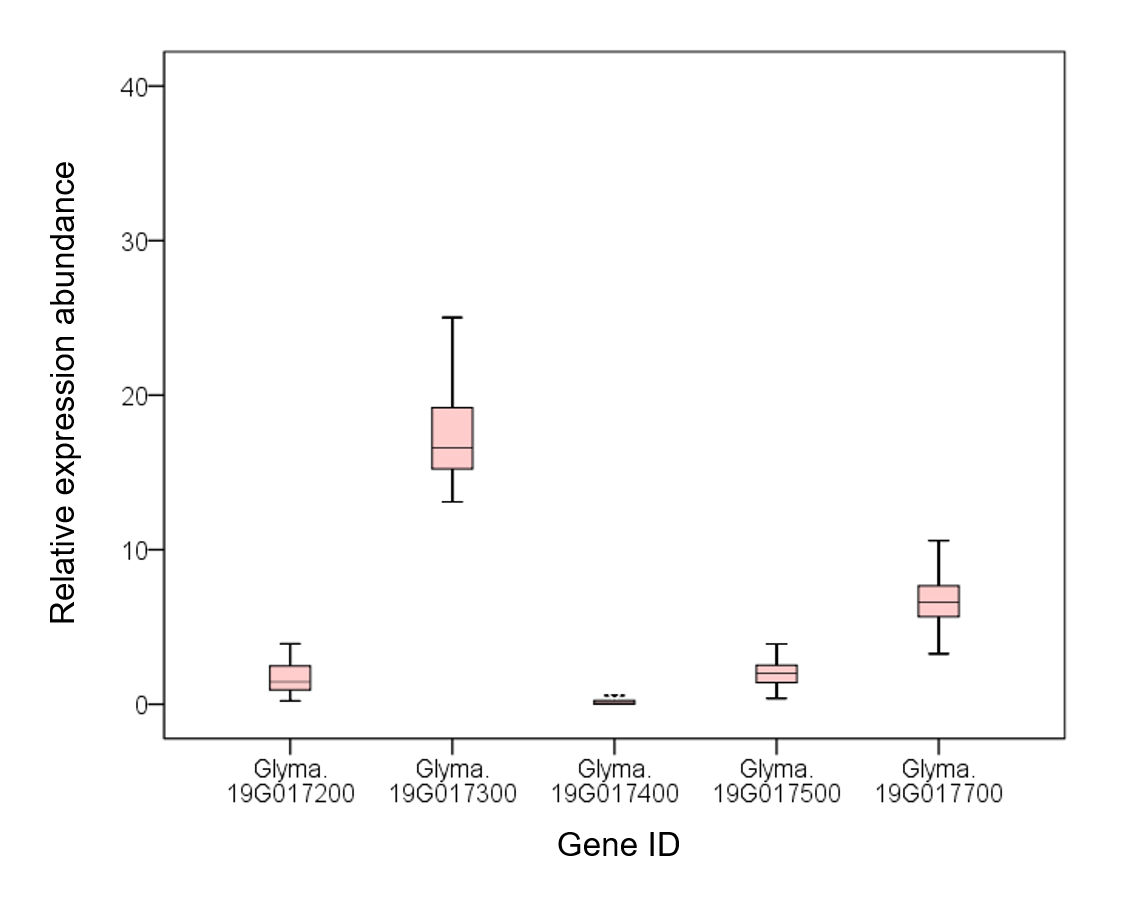


Figure S4 Relative expression abundance of the five candidate genes at the late R6 stage across 43 soybean accessions.


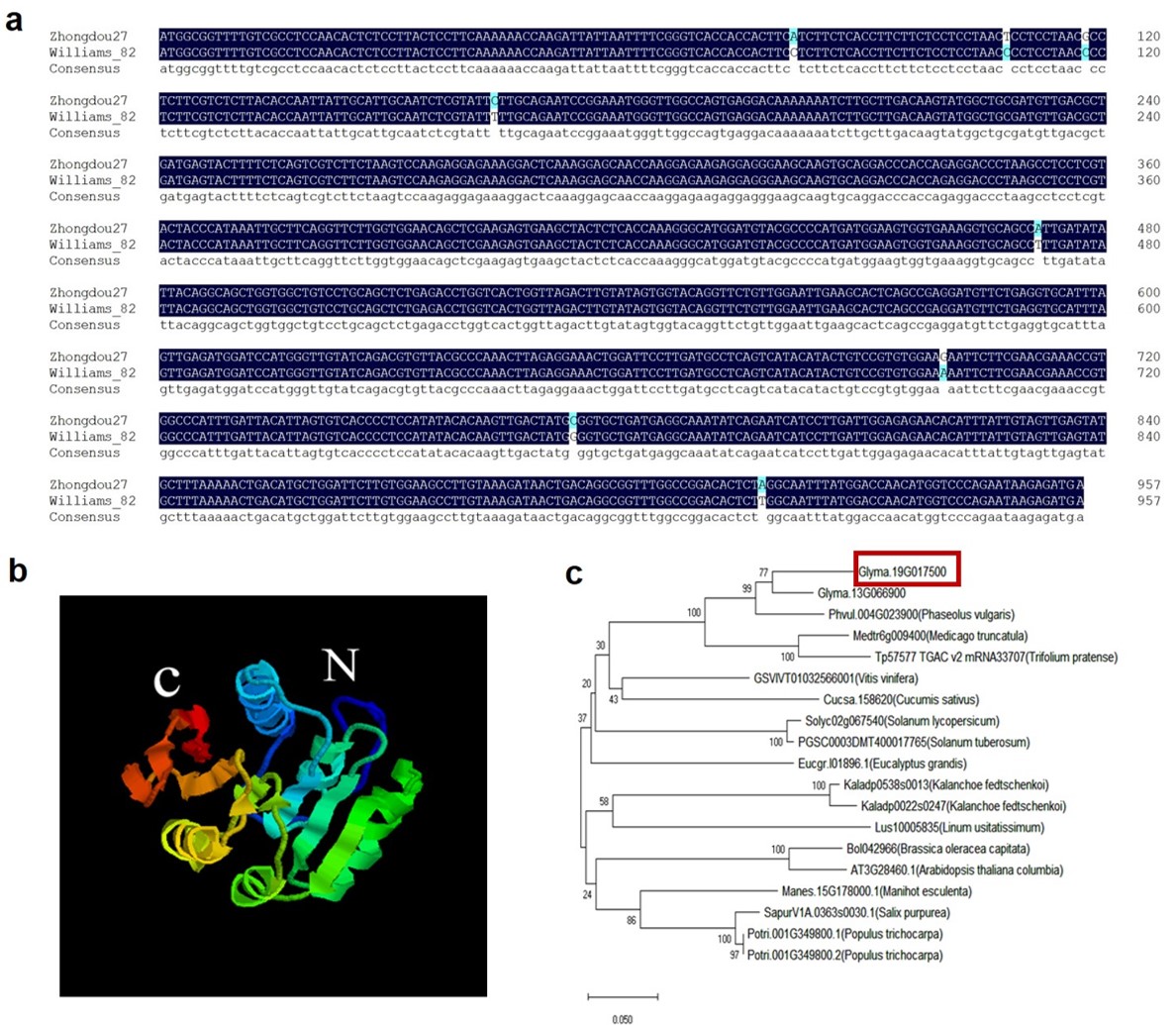


Figure S5 *GmMT1* cloning and sequence analysis. (a) Alignment of *GmMT1* from high-isoflavone soybean cultivar ‘Zhongdou27’ and from the reference genome (*Glycine max* Williams 82). (b) Predicted 3-D structure of the GmMT1 protein. (c) Phylogeny showing the methyltransferase proteins from 15 plant species. GmMT1 is boxed in red. Numbers at the nodes represent confidence level; scale bar represents evolutionary distance.


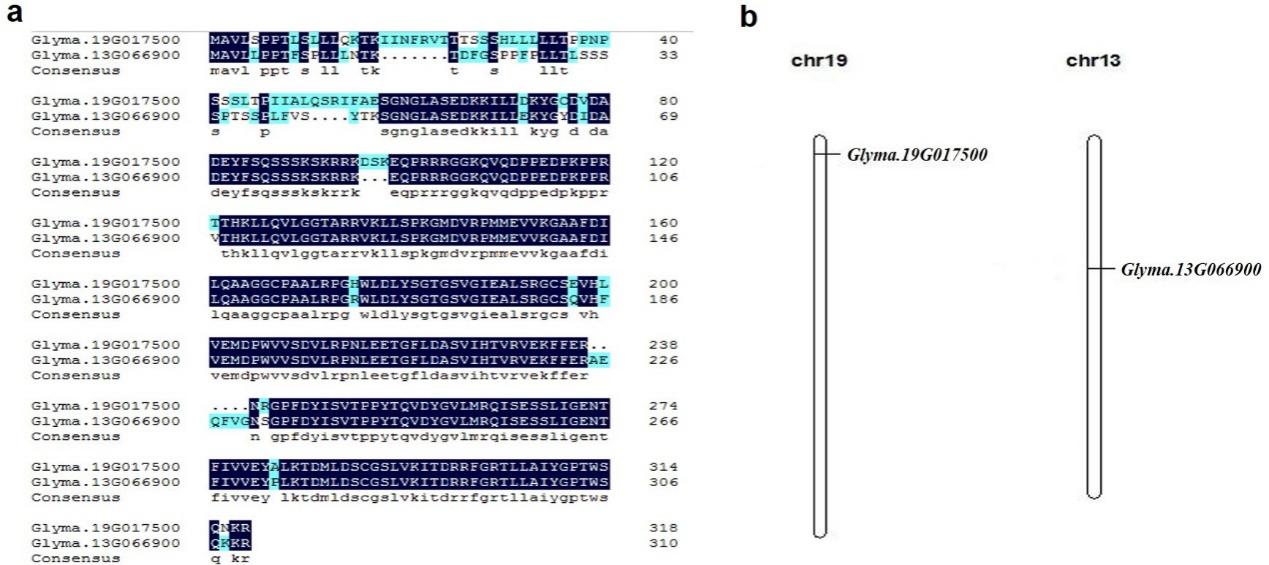


Figure S6 Identification of a homolog of *GmMT1* on chromosome 13*.* (a) Alignment of *GmMT1* and its homolog (*Glyma.13G066900*). (b) The chromosomal locations of *GmMT1* and *Glyma.13G066900*.


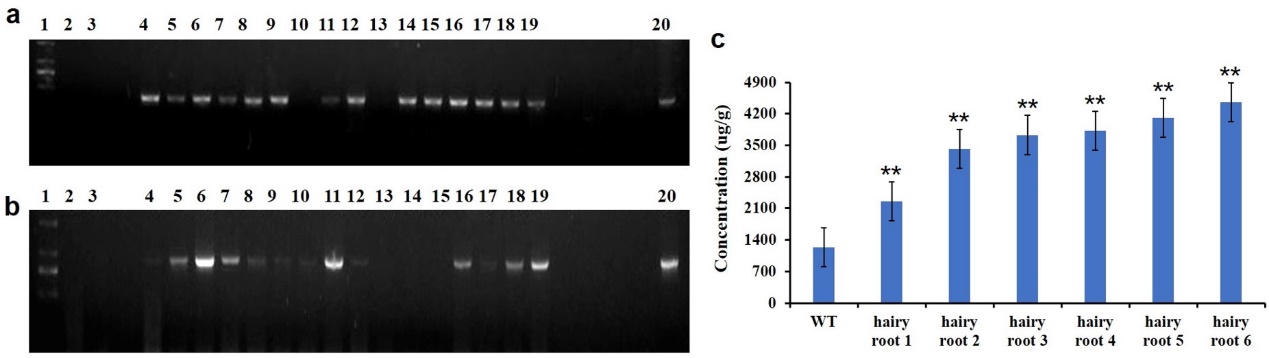


Figure S7 Overexpression of *GmMT1* in the hairy roots of soybean cultivar Donong50, a low-isoflavone cultivar. (a–b) PCR identification of transgenic roots based on the expression of (a) the marker gene *Bar* or (b) the *GmMT1*-gene-specific primer with a partial 35S promoter sequence. A total of 12 plants were positively detected by both primers simultaneously. (c) Isoflavone contents in the hairy roots of wild-type Donong50 and of six plants overexpressing *GmMT1* (randomly selected from among the 12 transgenic plants identified in panel a); three repeated measurements were made per plant*.* ** indicates a significant difference between WT and transgenic plants (P < 0.01, Student’s *t* test). Error bars indicate standard error (n = 3).


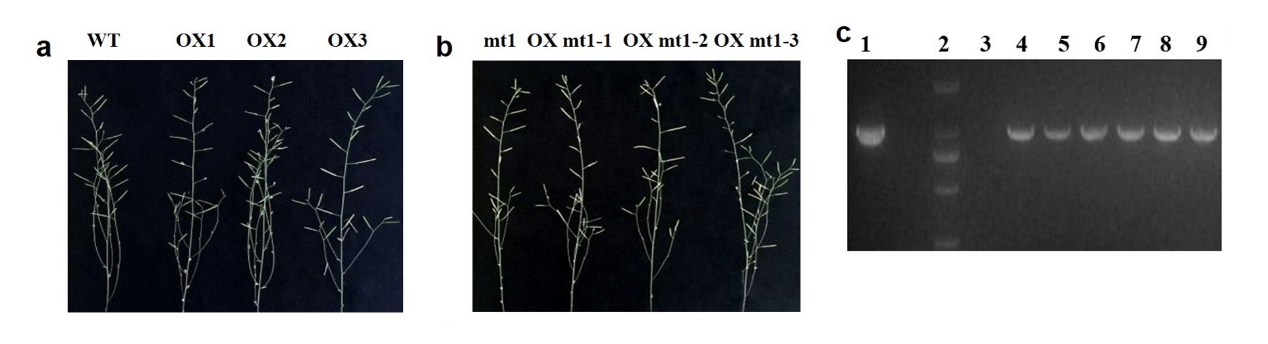


Figure S8 Transgenic *Arabidopsis* identification and trait evaluation. (a) Phenotypes of wild-type (WT) and *GmMT1-*overexpressing *Arabidopsis thaliana* Columbia-0 (Col-0) lines (OX1–3). (b) Phenotypes of *A. thaliana mt1,* a mutant strain in which the *Arabidopsis GmMT1* homolog (*At3G28460*) is silenced (mt1), and *GmMT1-*overexpressing strains of this mutant (OX mt1-1–3). (c) PCR identification of T3 *Arabidopsis* lines.

Table S1 The source and isoflavone content of 43 soybean accessions.

| Name | Country | Latitude | Longitude | Total isoflavone content (μg/g) |
| --- | --- | --- | --- | --- |
| Charleston | America | 41.83 | 92.91 | 5274.15 |
| Peking | America | 40.42 | 74.00 | 5090.55 |
| Baichengmoshidou | China | 43.35 | 126.29 | 4607.88 |
| Datunxiaohei | China | 39.19 | 116.29 | 5970.22 |
| HF47 | China | 48.29 | 128.08 | 3882.84 |
| Heersong2 | Ukraine | 50.28 | 30.29 | 4413.88 |
| HN55 | China | 48.29 | 128.08 | 3942.46 |
| Jinshanchamoshidou | China | 43.35 | 126.29 | 5156.93 |
| Jiunong21 | China | 43.35 | 126.29 | 3845.67 |
| Qinganhei | China | 48.29 | 128.08 | 4466.68 |
| Tie50 | China | 40.85 | 122.00 | 4361.32 |
| Xiaolimoshi | China | 48.29 | 128.08 | 3709.00 |
| Zhong6 | China | 40.15 | 116.28 | 3990.10 |
| Zhong03-5334 | China | 40.15 | 116.28 | 4448.65 |
| Zhong03-5373 | China | 40.15 | 116.28 | 4048.60 |
| Zhong95-5388 | China | 40.15 | 116.28 | 5225.84 |
| ZhongJ4033 | China | 40.15 | 116.28 | 4031.71 |
| DN L-10 | Canada | 43.40 | 79.25 | 4885.61 |
| Jiunong20 | China | 43.35 | 126.29 | 4699.19 |
| Zhong27 | China | 40.15 | 116.28 | 5969.79 |
| Huangbaodoyu | China | 40.85 | 122.00 | 1907.50 |
| DN L-79 | Canada | 43.40 | 79.25 | 1772.29 |
| Aika166 | Rumania | 44.23 | 26.10 | 1912.72 |
| B1873 | China | 48.29 | 128.08 | 1778.44 |
| Chasedou | China | 43.35 | 126.29 | 1801.45 |
| DN47 | China | 48.29 | 128.08 | 1851.79 |
| DN49 | China | 48.29 | 128.08 | 1957.33 |
| Duludou | China | 43.35 | 126.29 | 1848.94 |
| Ha04-1824 | China | 48.29 | 128.08 | 2042.51 |
| Heihehuang | China | 48.29 | 128.08 | 1716.84 |
| Hu04-528 | China | 44.49 | 111.70 | 1510.21 |
| Yuanbaojin | China | 48.29 | 128.08 | 1747.11 |
| HJ4403 | China | 48.29 | 128.08 | 1739.16 |
| KB1 | China | 48.29 | 128.08 | 1749.81 |
| Ken04-8579 | China | 48.29 | 128.08 | 1949.19 |
| Meng9 | China | 44.49 | 111.70 | 1263.64 |
| Silihuang | China | 48.29 | 128.08 | 1651.30 |
| Sui25 | China | 48.29 | 128.08 | 1959.40 |
| Sui28 | China | 48.29 | 128.08 | 1924.00 |
| Yapiche | China | 48.29 | 128.08 | 1877.57 |
| Sui29 | China | 48.29 | 128.08 | 1955.96 |
| Fengshou6 | China | 48.29 | 128.08 | 1505.95 |
| HJ2 | China | 48.29 | 128.08 | 1773.78 |

Table S2 SSR primers used for the fine-mapping of QTL qISO19-1.

| Marker Name | Maker ID | Chromosome | Daidzein LOD score | Total isoflavone LOD score | | motif | Forward primer | Reverse primer |
| --- | --- | --- | --- | --- | --- | --- | --- | --- |
| M1 | BARCSOYSSR_19_0132 | Gm19 | 0.147 | | 0.7864 | (TAT)17 | GGTCCACATGAAATGAAGGT | TCTCAGCCTGCAAAGAAAA |
| M2 | BARCSOYSSR_19_0101 | Gm19 | 0.1513 | | 0.8093 | (TA)11 | TTTTCAAAACAATCCGTCCC | GGGATGGTTATGAGTTGTGTCA |
| M3 | BARCSOYSSR_19_0105 | Gm19 | 1.1513 | | 0.8093 | (TA)25 | TCATGATTAGTGAGGTGAACCC | TCGGACAAAACTAAGTGTGTATGG |
| M4 | BARCSOYSSR_19_0116 | Gm19 | 3.4948 | | 3.2167 | (ATT)22 | CAAAAAGAAGAGAGAAAGTCCCA | CACCAAATAAAAATATTTGCTTTAGAA |
| M5 | BARCSOYSSR_19_0123 | Gm19 | 6.0405 | | 3.8778 | (AAT)25 | TCACACCTAACAAAGTAATGGTAGA | AAATAGGAAATTCTTAATGTGGATAAA |
| M6 | BARCSOYSSR_19_0125 | Gm19 | 2.9733 | | 2.8745 | (AT)11 | TTTTTGGAATGCGAAAATATTAAG | TCAAAATTTATATCTGGTTTTGCATT |
| M7 | BARCSOYSSR_19_0119 | Gm19 | 1.0821 | | 0.4473 | (AT)19 | TCAATAAAATGCGTATGCAACA | GAACATGAGTTTGATTTTCACCA |
| M8 | BARCSOYSSR_19_0110 | Gm19 | 1.1373 | | 0.7337 | (AAT)21 | CCGCATAAAAAACACAACAAATTA | GCGGGCAAATTTGACCTAACTCACAAC |
| M9 | BARCSOYSSR_19_0130 | Gm19 | 1.4433 | | 1.3109 | (TA)23 | TGGATCACTTTAAGGTGCTGAG | TCAGGAGCAGTGCATTTGTA |
| M10 | BARCSOYSSR_19_0135 | Gm19 | 0.2847 | | 1.496 | (TA)19 | TGATATGAATCCGTCCCAGT | GGCTAAGCACATGAACGTGA |
| M11 | BARCSOYSSR_19_0138 | Gm19 | 0.301 | | 1.5796 | (TA)14 | TTTGGTGACATGGCAAACAT | TCAAGTAAATGCTCATGCCAA |
| M12 | BARCSOYSSR_19_0142 | Gm19 | 0.443 | | 2.0078 | (AT)16 | TGGACCGCCATAAAAAGATT | CACCTTTCCCTTGGTCAAAA |

Table S3. Primer sequences used for PCR and qRT-PCR.

| Primer name | Sequences (5'-3') | Product length (bp) |
| --- | --- | --- |
| GmActin4-F | GTTTCAAGCTCTTGCTCGTAATCA | 214 |
| GmActin4-R | GTGTCAGCCATACTGTCCCCATTT |  |
| GmMT1-F | GAAGATCTATGGCGGTTTTGTCGCCT | 971 |
| GmMT1-R | CACGTGTCATCTCTTATTCTGGGACCATGTT |  |
| qGmMT1-F | CCCCTCTTCGTCTCTTACACC | 122 |
| qGmMT1-R | CGTCAACATCGCAGCCATAC |  |
| scGmMT-F | CGGGGGACTCTTGACATGGCGGTTTTGTCGCCTCC | 984 |
| scGmMT-R | GTCAGATCTACCATGCTCTTATTCTGGGACCATGT |  |
| 3301-bar-F | TGCACCATCGTCAACCACTACATC | 433 |
| 3301-bar-R | GCTGCCAGAAACCCACGTCAT |  |
| 3301-35S-GmMT1-F | TGGCGAACAGTTCATACAGA | 789 |
| 3301-35S-GmMT1-R | GGTTGCTCCTTTGAGTCCTT |  |

Table S4 Isoflavone contents in the parent populations (‘Zhongdou27’ and ‘Dongnong8004’) grown in two different locations.

| Trait | Location | Zhongdou27 | Jiunong20 | Mean ± SD^a^ (µg/g) | Range (µg/g) | Kurtosis | Skewness | CV^b^ |
| --- | --- | --- | --- | --- | --- | --- | --- | --- |
|  |  | (µg/g) | (µg/g) |  |  |  |  |  |
| DZ | e1 | 2287.79 | 929.92 | 1384.01±364.25 | 712.79–3342.32 | 2.12 | 1.28 | 0.35 |
| DZ | e2 | 2471.06 | 871.67 | 1171.25±315.03 | 430.36–3138.52 | −0.13 | 0.73 | 0.43 |
| GC | e1 | 208.83 | 112.55 | 317.63±47.554 | 212.33–479.97 | 3.12 | 1.33 | 0.18 |
| GC | e2 | 217.52 | 192.68 | 302.6±44.3 | 193.44–424.87 | 2.82 | 1.28 | 0.16 |
| GT | e1 | 2288.05 | 640.73 | 1188.06±368.52 | 564.37–2916.15 | 2.82 | 1.43 | 0.33 |
| GT | e2 | 2424.92 | 544.55 | 1119.02±316.07 | 489.60–2998.02 | −0.3 | 0.38 | 0.41 |
| TI | e1 | 4784.67 | 1683.2 | 2889.69±690.39 | 1495.10–6671.87 | 3.52 | 1.57 | 0.3 |
| TI | e2 | 5113.5 | 1608.9 | 2592.89±605.53 | 1285.93–6466.23 | 2.9 | 1.47 | 0.37 |

^a^Standard error. ^b^Variable coefficient. e1 and e2 indicate the two experimental locations, ‘Xiangyang’ and ‘Hulan’, respectively.

Table S5 Resequencing statistics for the two parental lines (‘Zhongdou27’ and ‘Dongnong8004’) and the 119 recombinant inbred lines (RILs) in the mapping population.

| Sample | Reads (M) | Bases (G) | GC (%) | Q20 | Sequencing depth | Mapped reads (%) | Coverage (%) |
| --- | --- | --- | --- | --- | --- | --- | --- |
| Zhongdou27 | 156.5 | 26.18 | 35.7 | 97.58 | 24.54 | 78.88 | 94.62 |
| Dongnong8004 | 145.56 | 25.58 | 35.84 | 97.56 | 22.86 | 78.3 | 94.53 |
| Average across the RILs | 33.08 | 3.46 | 36.48 | 97.54 | 3.65 |  |  |

Table S6 Genetic map information for the RIL population, derived by crossing ‘Zhongdou27’ and ‘Dongnong8004’.

| Chromosome | Bin marker number | Length of linkage group (cM) |
| --- | --- | --- |
| Chr.1 | 152 | 91.83 |
| Chr.2 | 166 | 132.71 |
| Chr.3 | 171 | 94.04 |
| Chr.4 | 115 | 92.43 |
| Chr.5 | 115 | 101.11 |
| Chr.6 | 199 | 123.86 |
| Chr.7 | 138 | 117.00 |
| Chr.8 | 90 | 94.01 |
| Chr.9 | 180 | 101.14 |
| Chr.10 | 138 | 132.01 |
| Chr.11 | 119 | 107.61 |
| Chr.12 | 88 | 90.58 |
| Chr.13 | 225 | 131.27 |
| Chr.14 | 125 | 104.31 |
| Chr.15 | 90 | 132.73 |
| Chr.16 | 94 | 108.65 |
| Chr.17 | 87 | 166.63 |
| Chr.18 | 161 | 89.40 |
| Chr.19 | 97 | 85.44 |
| Chr.20 | 97 | 76.22 |
| Total | 2647 | 2172.98 |

Table S7 Genes in the fine-mapping interval of qISO19-1, based on comparisons with the reference genome.

| Gene ID | Start | End | Distance | Strand | Annotation |
| --- | --- | --- | --- | --- | --- |
| Glyma.19G017200 | 1785304 | 1799141 | 13837 | − | Sugar isomerase (SIS) family protein |
| Glyma.19G017300 | 1814297 | 1816879 | 2582 | + | Leucine-rich repeat protein kinase family protein |
| Glyma.19G017400 | 1819967 | 1821863 | 1896 | − | CLAVATA3/ESR-RELATED 25 |
| Glyma.19G017500 | 1827038 | 1830895 | 3857 | − | Methyltransferases |
| Glyma.19G017700 | 1838597 | 1843202 | 4605 | + | Protein of unknown function (DUF1336) |

Table S8 Correlation between the relative expression levels of the five candidate genes and total isoflavone contents at late R6 stage in 43 soybean germplasms.

|  | Expression abundance in soybean seeds at the R7 stage | | | | |
| --- | --- | --- | --- | --- | --- |
|  | Glyma19G017200 | Glyma19G017300 | Glyma19G017400 | Glyma19G017500 | Glyma19G017700 |
| Total isoflavone content | −0.09 |  |  |  |  |
|  |  | −0.16 |  |  |  |
|  |  |  | −0.31 |  |  |
|  |  |  |  | 0.64^**^ |  |
|  |  |  |  |  | 0.25 |

** indicates a significant correlation (P < 0.01).
